# Temozolomide, sirolimus and chloroquine is a new therapeutic combination that synergizes to disrupt lysosomal function and cholesterol homeostasis in GBM cells

**DOI:** 10.18632/oncotarget.23855

**Published:** 2018-01-03

**Authors:** Sanford P.C. Hsu, John S. Kuo, Hsin-Chien Chiang, Hsin-Ell Wang, Yu-Shan Wang, Cheng-Chung Huang, Yi-Chun Huang, Mau-Shin Chi, Minesh P. Mehta, Kwan-Hwa Chi

**Affiliations:** ^1^ Department of Neurosurgery, Neurological Institute, Taipei Veterans General Hospital, Taipei, Taiwan; ^2^ School of Medicine, National Yang Ming University, Taipei, Taiwan; ^3^ Department of Neurological Surgery, School of Medicine and Public Health, University of Wisconsin, Madison, WI, USA; ^4^ JohnPro Biotech Inc., Taipei, Taiwan; ^5^ Department of Biomedical Imaging and Radiological Sciences, National Yang-Ming University, Taipei, Taiwan; ^6^ Department of Radiation Therapy and Oncology, Shin Kong Wu Ho-Su Memorial Hospital, Taipei, Taiwan; ^7^ Miami Cancer Institute, Miami, FL, USA

**Keywords:** autophagy, rapamycin, chloroquine, lysosome cell death, cholesterol

## Abstract

Glioblastoma (GBM) cells are characterized by high phagocytosis, lipogenesis, exocytosis activities, low autophagy capacity and high lysosomal demand are necessary for survival and invasion. The lysosome stands at the cross roads of lipid biosynthesis, transporting, sorting between exogenous and endogenous cholesterol. We hypothesized that three already approved drugs, the autophagy inducer, sirolimus (rapamycin, Rapa), the autophagy inhibitor, chloroquine (CQ), and DNA alkylating chemotherapy, temozolomide (TMZ) could synergize against GBM. This repurposed triple therapy combination induced GBM apoptosis *in vitro* and inhibited GBM xenograft growth *in vivo*. Cytotoxicity is caused by induction of lysosomal membrane permeabilization and release of hydrolases, and may be rescued by cholesterol supplementation. Triple treatment inhibits lysosomal function, prevents cholesterol extraction from low density lipoprotein (LDL), and causes clumping of lysosome associated membrane protein-1 (LAMP-1) and lipid droplets (LD) accumulation. Co-treatment of the cell lines with inhibitor of caspases and cathepsin B only partially reverse of cytotoxicities, while N-acetyl cysteine (NAC) can be more effective. A combination of reactive oxygen species (ROS) generation from cholesterol depletion are the early event of underling mechanism. Cholesterol repletion abolished the ROS production and reversed the cytotoxicity from QRT treatment. The shortage of free cholesterol destabilizes lysosomal membranes converting aborted autophagy to apoptosis through either direct mitochondria damage or cathepsin B release. This promising anti-GBM triple therapy combination severely decreases mitochondrial function, induces lysosome-dependent apoptotic cell death, and is now poised for further clinical testing and validation.

## INTRODUCTION

GBM is an aggressive malignancy with high mortality, and relative resistance to radiation and other treatments [1, 2]. Current treatments include maximal surgery followed by adjuvant radiation (RT) and chemotherapy (CT) with TMZ [3]. The addition of bevacizumab to standard RT-TMZ therapy did not improve overall survival. Pre-clinical data suggest that this failure may be due to tumor metabolic adaptation toward anaerobic metabolism, and increased tumor cell invasiveness after anti-angiogenic treatment [4, 5]. These represent examples of adaptive stress-response by the tumor and therefore therapies that induce and amplify tumor stress response, such as autophagy, unfolded protein response (UPR) and cancer metabolism, represent potential therapeutic strategies.

GBM is characterized by exaggerated lipogenesis, enhanced LDL cholesterol uptake, high phagocytosis and micro-vesicle exocytosis activities, and depends very much on cholesterol homeostasis for constant membrane changes. The majority of cholesterol is found at the plasma membrane, enriched as raft micro-domain, while the endoplasmic reticulum (ER), mitochondria and lysosomes contain minimal amount of cholesterol. The lysosome is on the end point of endocytic, phagocytic and autophagocytic pathway and responsible for their cellular trafficking and play the key role in maintain cholesterol homeostasis. Because of blood brain barrier, GBM cells constantly require cholesterol from tumor microenvironment (TME), its high lysosomal demand may be an “Achilles’ heel”, an exploitable vulnerability of GBM [6, 7]. Recently, Villa *et al.* also showed that GBM cells are dependent on cholesterol for survival, and succumb to Liver X receptor (LXR) agonist-induced cholesterol depletion by decreased influx and increased efflux of cholesterol [8]. GBM cells can convert excessive cholesterol to cholesterol ester for storage in LD, which were mobilized to fuel fatty acid oxidation to sustain cell viability during nutrient deprivation stress [9]. The suppression of LD formation or the *de novo* cholesterol synthesis all had been reported to suppress GBM growth [10, 11]. We try to disturb the Achilles’ heel with autophagy modulation.

Compared to lower grade gliomas, GBMs express lower intrinsic autophagy activity, whereas higher expression of autophagy genes, LC3 and Beclin1 correlates with better survival [12]. High grade glioma has insufficient autophagy due to promoter hyper-methylation and downregulation of the autophagy inducer ULK2, resulting in tumor growth [13]. Lysosomes are primarily involved in the degradation, recycling and secretory pathways for nutrient homeostasis [14]. All of the inter-connections between membrane and intracellular organelles require cholesterol homeostasis. Intracellular cholesterol trafficking is very important for normal ER and lysosome functions [15]. Lower tumor autophagy activity is compensated with higher tumor phagocytic activity to obtain nutrition from TME and higher lysosomal exocytosis for clearance of ER oxidative stress [16]. We hypothesized that cholesterol trafficking between the plasma membrane and intracellular compartments is also a required cellular process that could also be disrupted by the paradoxical combination of autophagy inducers (rapamycin, Rapa) and inhibitors (Chloroquine, CQ). Synergistic effects of this paradoxical combining had reported work in many tumors such as melanoma, bladder cancer, colon cancer, hepatoma, sarcoma and glioma [17–23]. In general, the double combination may not be lethal unless elevated ER stress is also present. Therefore, we proposed and started testing a new “triple combination” therapeutic strategy against a variety of cancers by combining the autophagy inducer/inhibitor regimen with chemo- or radiotherapy to induce ER stress. We have reported clinical safety and promising efficacy for this triple combination strategy in a variety of cancer patients including GBM patients, who received standard of care TMZ plus RT (that induce ER stress) with CQ and Rapa [24, 25].

The stress from TMZ treatment combined with mTOR inhibition with Rapa treatment increases autophagy demand, but CQ inhibits lysosome degradation and the aborted autophagy increased the mitochondria burden. CQ plus Rapa treatment decreases the glycolytic and oxygen consumption rates, and inhibits the conversion of cholesterol ester to free cholesterol and lipolysis from LD resulting in cholesterol depletion destabilizes lysosomes and causing cathepsin release. LD accumulation is a sign of impaired mitochondria function [9]. The shortage of fatty acid for β-oxidation resulted in energy crisis and further potentiates the mitochondria burden and oxidation damage and converts tumor cells to apoptosis.

## RESULTS

### CQ, Rapa and TMZ combination treatment synergistically increase apoptotic cell death, inhibits cell proliferation in GBM cell lines

**Figure 1:**
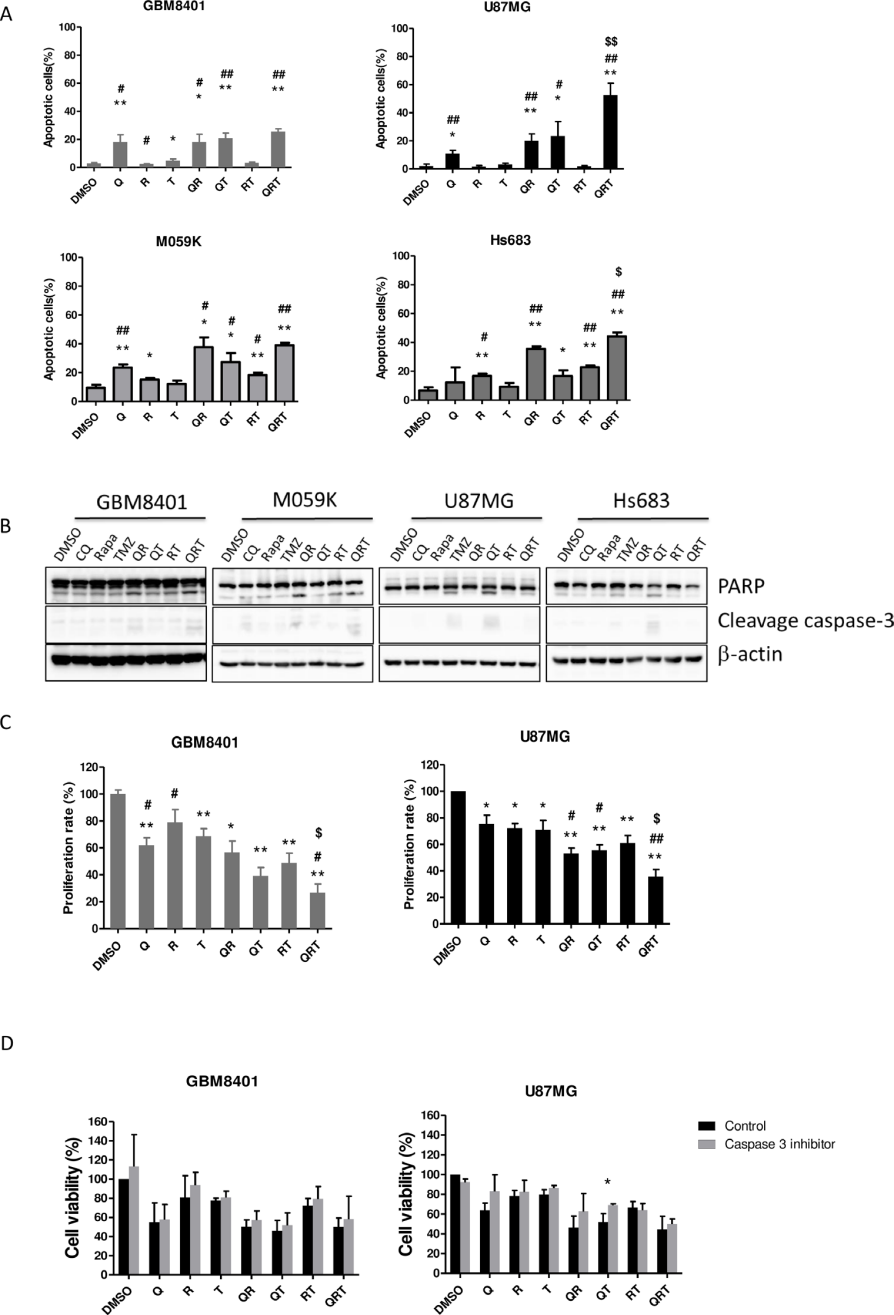
Effect of CQ (Q), Rapa (R) and TMZ (T) in different combination treatment on apoptosis and proliferation in 4 GBM cell lines All GBM cell lines were cultured as following treatment to assay apoptotic cells (GBM8401 Q 40/R 12.5/T 400 μM 24 h, M059K Q90/R 20/T 400 μM 24 h, U87MG Q 40/R 10/T 400 μM 48 h, Hs683 Q 15/R 20/T 400 48 h. (**A**) The population of annexin V+ apoptotic cells was evaluated by flow cytometry using annexin V-FITC/PI staining in GBM8401 cells, M059K, U87MG and Hs683 cells after CQ, Rapa and TMZ treatment. (**B**) The expression of cleaved PARP and cleavage caspase-3 in GBM8401 M059K, U87MG and Hs683 cells were analyzed by western blotting. (**C**) Cell proliferation in GBM8401 cells and U87MG cells after CQ, Rapa and TMZ treatment was assessed by MTS assay. The Y-axis represents the proliferation rate, calculated as the ratio to control untreated cells. The graphs shown represent the mean ± SE of at least three different experiments. (**D**) All GBM cell lines were treated CQ, Rapa, and TMZ in different combination following 1 h caspase-3 inhibitor (z-VAD-FMK) pretreatment and then assessed for cell viability. Statistical significance of CQ or Rapa or TMZ or combinations *vs* DMSO is indicated (^*^). Statistical significance of CQ or Rapa or combinations *vs* TMZ alone is indicated (^#^). Statistical significance of QRT *vs* QR is indicated (^$^). Statistical significance: ^*^P < 0.05, ^**^P < 0.01; ^#^*P* < 0.05, ^##^*P* < 0.01, ^$^*P* < 0.05.

We investigated the effect of CQ (Q), Rapa (R) and TMZ (T) combination treatment on cellular apoptosis and viability in 4 GBM cells lines. As shown in Figure 1A, induction of apoptotic cell death is most significant with the presence of CQ in all combinations (Q, QR, QT and QRT), slightly in RT treatment, but not with R, and T treatments. QRT treatment resulted in the highest level of apoptotic cell death in all GBM cell lines. In apoptotic cell death, a clear difference emerged in the pattern of cell death across the GBM cell lines, dividing the cell lines into two groups. Group I cells, GBM8401 and M059K displayed less sensitivity to TMZ treatment, QR treatment was pretty toxic to them and were not induced more apoptotic cell death after QRT treatment (Figure 1A). Group II cells, U87MG and Hs683 showed more sensitive to TMZ treatment. QRT treatment significantly increased apoptosis comparison with QR and QT treatment. Expression of PARP and cleavage caspase-3 were showed in QR/QRT treatment of Type-I GBM cell lines and T/QT treatment of Type-II GBM cell lines (Figure 1B). Hypermethylation of the O6-methylguanine-DNA- methyltransferase (MGMT) gene has been shown to be associated with better response to TMZ. However, the MGMT promoter methylation status in these GBM cell lines were not associated with TMZ sensitivity (Supplementary Figure 1A). We, therefore, chose GBM8401 and U87MG as representative of these two types for further studies. The QRT combination significantly inhibited cell proliferation especially on U87MG cells compared with GBM8401 cells (Figure 1C). Results of dose-dependent experiments were present in supplementary results (Supplementary Figure 1B). Pre-treatment with caspase 3 inhibitor z-VAD-FMK (10 uM treatment for 1 h) can only partially reverse the cytotoxicity in QT treated U87MG (Figure 1D) but not others. Consequently, QRT synergism were caspase-3 independent in GBM8401 and U87MG cell lines.

**Figure 2:**
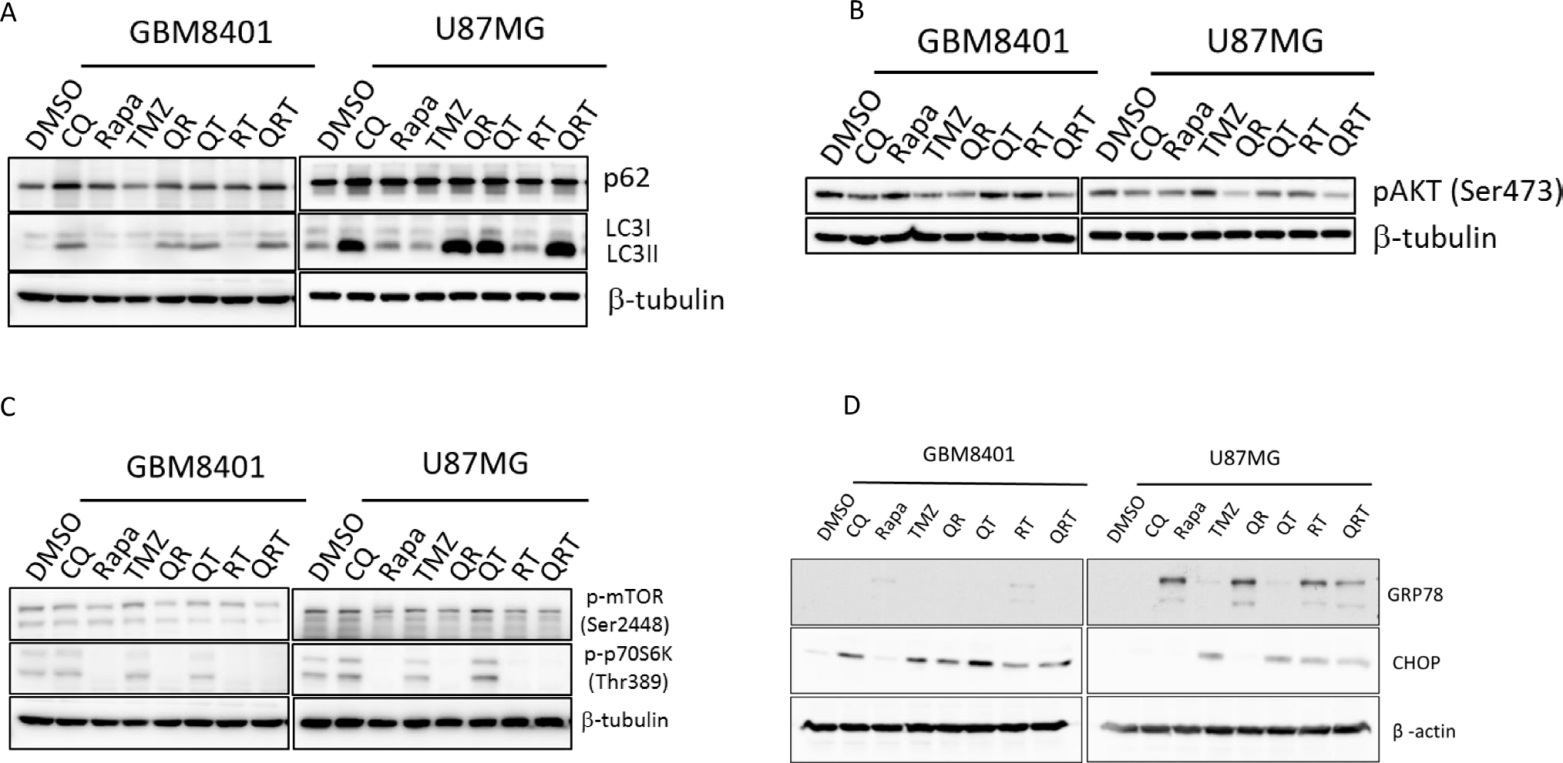
Autophagy, ER stress, and p-AKT change after CQ (Q), Rapa (R) and TMZ (T) in different combination treatment GBM8401 cells and U87MG cells were treated with CQ, Rapa and TMZ in different combinations, harvested at 24 hours and immunoblotted for LC3-I, LC3-II and (**A**) p62, (**B**) p-AKT, (**C**) p-mTOR and p-S6K, (**D**) GRP78 and CHOP.

Both cell lines had basal LC3II and p62 expression. Accumulation of LC3II and p62 universally increased with addition of CQ, suggesting that both cell lines are moderately sensitive to autophagy inhibition (Figure 2A). In GBM8401 and U87MG, QR and QRT treatment decreased p-Akt activity more than with CQ alone or QT combination (Figure 2B). Rapamycin indeed inhibit p-p70S6k expression in both cell lines (Figure 2C). Interestingly, CHOP expression was only moderately increased after TMZ treatment with or without combination therapies, suggesting that ER stress does not significantly contribute to GBM cell death (Figure 2C and 2D). Rapa increased autophagy and decreased CHOP in both GBM lines, but GRP78 expression was only observed in U87MG cells after Rapa treatment, suggested a GRP78-induced autophagy process.

### CQ, Rapa and TMZ combination treatment induced oxidative stress and mitochondrial damage through energy crisis

**Figure 3:**
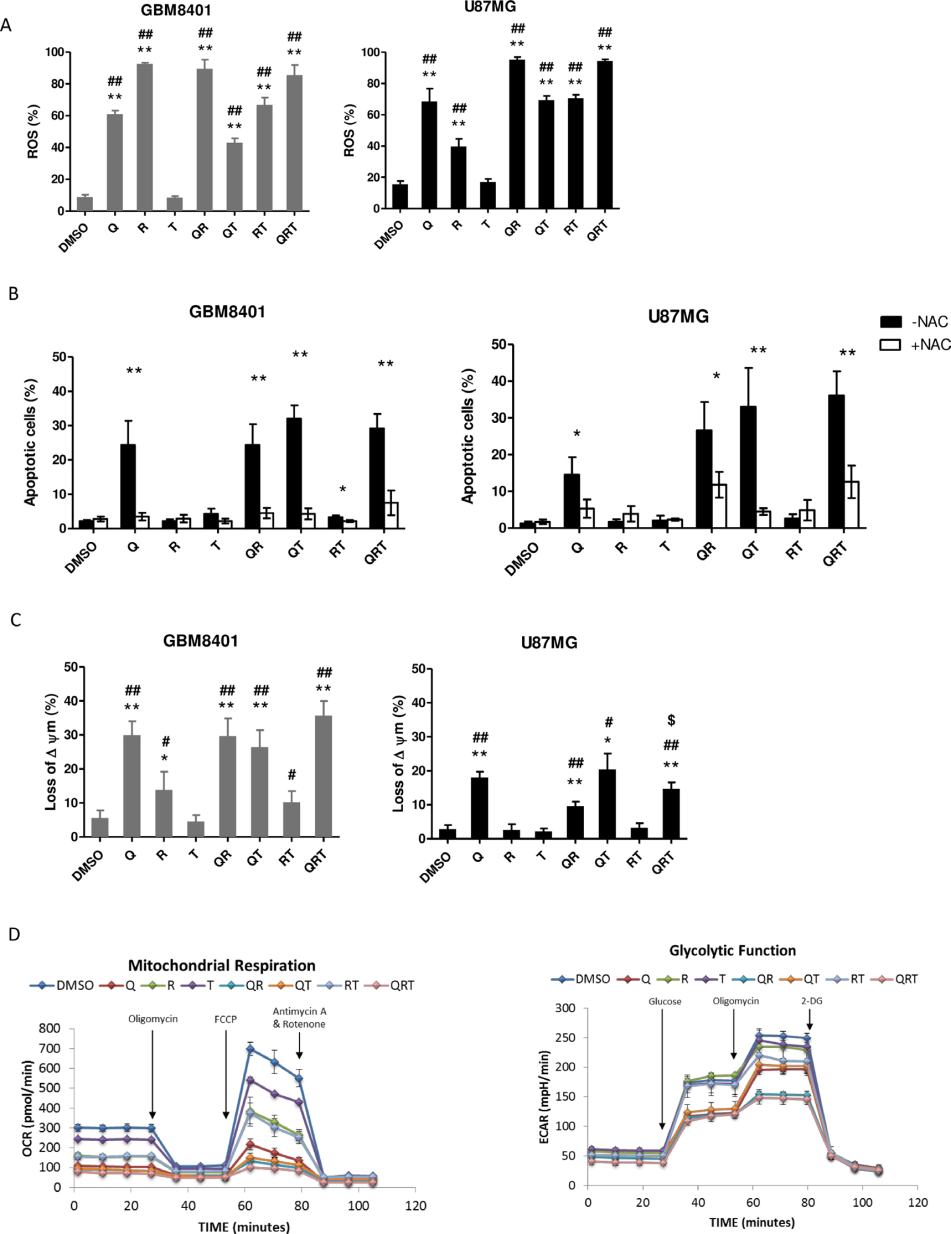
The metabolic change after CQ (Q), Rapa (R) and TMZ (T) in different combination treatment Cells were treated CQ (90 or 40 μM), Rapa (12.5 or 10 μM) and TMZ (400 μM) for 24 h or 48 h. (**A**) ROS generation in GBM8410 and U87MG were detected by BD Accuri™ C6 flow cytometer. Date were processed and analyzed with BD Accuri™ C6 software. GBM8401 and U87MG cells were treated with or without 20 mM NAC. (**B**) Apoptosis was assessed for analysis. (**C**) Loss of mitochondria membrane potential (MMP) was assessed via JC-1 staining and flow cytometry in GBM8401 and U87MG cells. The percentage of cells with low JC-1 fluorescence is shown. (**D**) Mitochondrial respiration and glycolytic capacity in GBM8401 Left panel: evaluation of OXPHOS capacity of GBM8401 cells were plated at 1 × 10^5^/well and cultured with each conditions for 2 h. Metabolic responses were evaluated after sequential injection of the following metabolic toxins: oligomycin, FCCP, antimycin A/rotenone. Right panel: evaluation of the glycolytic capacity of GBM8401 cultured with each conditions for 2 h. Metabolic responses were evaluated after sequential injection of the following metabolic toxins: Glucose, oligomycin and 2-DG. Statistical significance of CQ or Rapa or TMZ or combinations *vs* DMSO is indicated (^*^). Statistical significance of CQ or Rapa or combinations *vs* TMZ alone is indicated (^#^). Statistical significance of CQ or Rapa or TMZ or combinations *vs* QRT is indicated (^$^). Statistical significance: ^*^P < 0.05, ^**^P < 0.01; ^#^*P* < 0.05, ^##^*P* < 0.01, ^$^*P* < 0.05.

To investigate whether ROS induction plays a major role in cell death after treatment with QRT. We found treatment with Q, R alone or QT, QR or QRT markedly increased ROS generation except T (Figure 3A). Importantly, 20 mM NAC provided moderate to complete protection against Q, QR, QT, and QRT treatment in both GBM8401 and U87MG (Figure 3B), which indicated that ROS generation played early and imperial role in QR toxicities. Cells cultured with Q alone or QR, QRT treatments all showed significant loss of MMP (Figure 3C), Rapa alone increased ROS generation but did not lead to loss of MMP. GBM cells are characterized by high glycolytic activity and mitochondrial dysfunction [26]. Metabolic changes were investigated with the GBM8401 cell line. As shown in Figure 3D, the OCR was decreased with all combinations except TMZ alone (Figure 3D, left panel). QRT combination resulted in most severe OCR impairment, with almost complete elimination of mitochondrial function. In an ECAR assay, similar decreases in glycolytic function were observed with CQ, Rapa, RT, QT, with the most significant depression observed with the QRT combination (Figure 3D right panel). These results indicated that QRT treatment induced by oxidative stress on both cells while GBM8401 was more vulnerable to mitochondria induced metabolic catastrophe.

### CQ, Rapa and TMZ combination destabilizes lysosomes and abrogates lipid droplets breakdown

**Figure 4:**
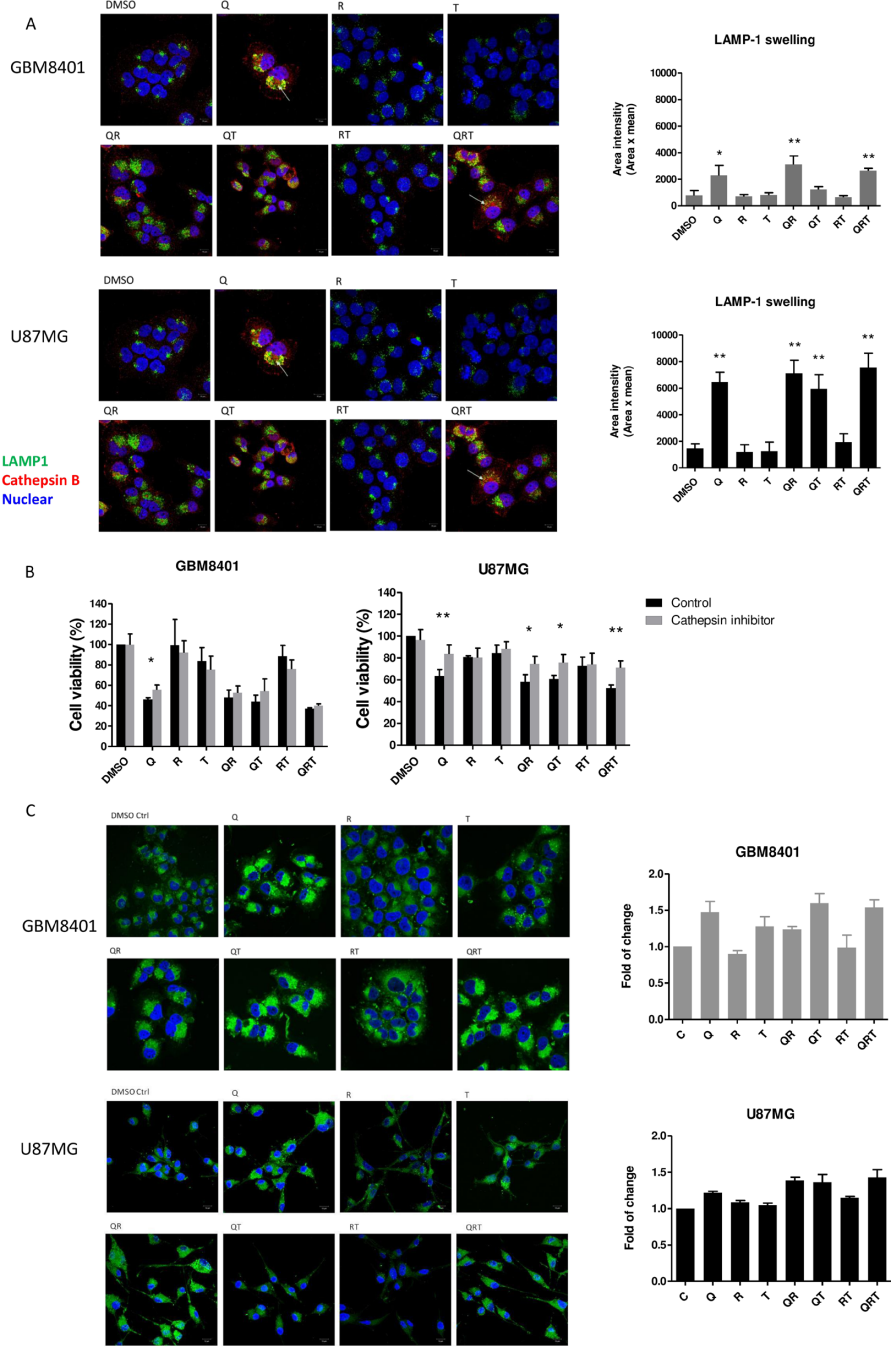
Lysosomal swelling and lipid droplets (LDs) accumulation after CQ (Q), Rapa (R) and TMZ (T) in different combination treatment (**A**) Immunostain with LAMP1 (a lysosome marker, green) and cathepsin B (red), quantified using Image J. (**B**) GBM cell lines were preincubated with cathepsin inhibitor (5 μM) for 1 h before the addition of CQ, Rapa and TMZ. Histograms show quantification of cell viability at day 1 (GBM8401) and day 2 (U87MG). (**C**) The change of LDs in U87MG and GBM8401 after CQ, Rapa and TMZ combination for 24 hrs. LDs was staining by BODIPY 493/503 (green) and Hoechst 33342 (nuclear, blue). Histograms were showed quantification of accumulation LDs by BODIPY 493/503 staining using flow cytometry. Statistical significance of CQ or Rapa or TMZ or combinations *vs* DMSO is indicated (^*^). Statistical significance: ^*^P < 0.05, ^**^P < 0.01. Scale bar: 10 μm.

Autophagy inhibition-mediated cellular apoptosis was recently reported to correlate with lysosomal cell death [27]. CQ is known to induce apoptosis by lysosomal membrane permeabilization, cathepsin release, and activation of BH3 interacting-domain death agonist (BID) and caspases [28, 29]. mTORC1 inhibitors such as Rapa decrease cholesterol biosynthesis and cause rapid lysosome cell death [23]. We investigated whether multiple autophagy modulations change lysosomal function. Figure 4A show abundant clumping and swelling of lysosomes with accumulation of LAMP-1 after Q, QR, QT and QRT treatment. The increased cathepsin B release from lysosomal swelling was suggested. Accordingly, we treated cathepsin inhibitor to observe whether cathepsin B dependent cell death played a major role. GBM8401 and U87MG were pretreated with cathepsin inhibitor for 1 h before treating them with Q, R, T and combination for 24 h. As shown in Figure 4B, in U87MG cells pretreated with cathepsin inhibitor, cell viability was significantly increase in Q, QR, QT and QRT treatment. However, GBM8401 cells were partially recovered cell viability in only Q treatment. These finding indicated that U87MG cells is more vulnerable to lysosome cell death, while as previously described, the GBM8401 deceased mainly by mitochondria cell death mechanism.

When free cholesterol increases, cholesteryl esters (CE) are formed and sequestered into LDs [30]. The breakdown of LDs is attributed to the actions of cytosolic hydrolytic enzymes or lipases. Recently, several studies have demonstrated an association between the lysosomal degradative pathway of autophagy (lipophagy) and the breakdown of intracellular LDs stores as survival mechanism [9, 31]. It was reported glioma cells accumulate LDs under hypoxic conditions, and this is directly correlated with the degree of malignancy and tumor growth [32]. To determine the change in LDs from baseline to post-treatment, GBM cells were visualized with fluorescent lipid dye BODIPY 493/503. In Figure 4C, LDs observed in GBM8401 cells slightly decreased after Rapa or RT treatment compared to untreated controls, suggesting that Rapa-induced lipophagy processes lead to the breakdown of LDs. On the contrary, CQ contributed to marked LDs accumulation from aborted lipophagy, and TMZ resulted in modest LD accumulation - possibly a resistance mechanism to hydrophobic drug [33].

### QRT treatment disturbs lipid droplet utilization and cholesterol homoeostasis

**Figure 5:**
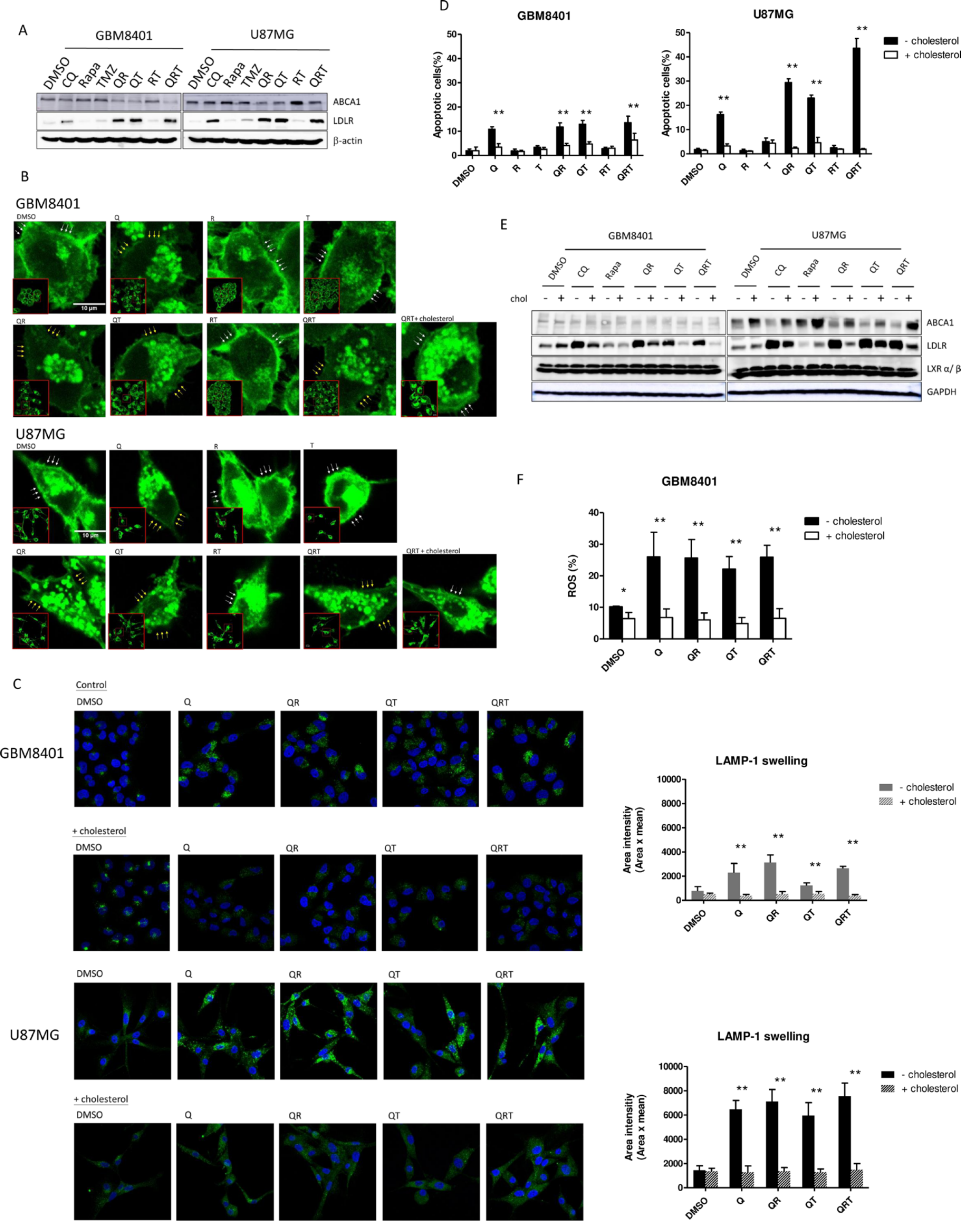
Changes of cholesterol homeostasis under CQ (Q), Rapa (R) and TMZ (T) in different combination treatment (**A**) The expression of ABCA1, LDLR after different drug combinations for GBM 8401 cells and U87MG cells. (**B**) Cholesterol stain assay. GBM8401 cells and U87MG cells were treated, fixed and stained with filipin to visualize cholesterol (green). High-magnification imaging reveals that addition of free cholesterol (10 μg/mL) regains partial cell membrane under QRT treatment (whit arrow line display rich cholesterol cell membrane; yellow arrow line display lacking cholesterol cell membrane). (**C**) Salvage treatment with free cholesterol and stained for LAMP-1 (green). Representative images are shown alongside the quantification of lysosome swelling. (**D**) The added of free cholesterol with different drug combinations could recover cell survival. (**E**) Decreased LDLR expression and increase ABCA1 expression was noted, but the expression of LXR were not change. (**F**) ROS production was decreased after free cholesterol in Q, QR, QT, QRT treatments in GBM8401 cells. Statistical significance is indicated (^*^). Statistical significance: ^*^P < 0.05, ^**^P < 0.01. Scale bars represent 10 μm.

We observed a marked increase in GBM expression of low-density lipoprotein receptor (LDLR) in response to the free cholesterol deficit after Q, QR, or QRT treatments (Figure 5A). CQ inhibits the conversion of endocytosed LDL to free cholesterol, and limited the lipophagy process from LD. The majority of the available free cholesterol is likely required for active cell membrane repair, to maintain cell membrane integrity because of increased exocytosis after triple combination treatment. The absence of ATP-binding cassette subfamiliy A membrane 1 (ABCA1), a cellular cholesterol efflux transport, overexpression after QRT triple combination treatment suggests that there was no associated efflux of cholesterol through this transporter (Figure 5A). We used filipin to visualize cellular cholesterol in three-drug treatment. The results show that decrease of membrane cholesterol in Q, QR, QT and QRT treatment in both cell lines and cholesterol treatment rescues partial membrane cholesterol in QRT treatment (Figure 5B). The data showed that Q, T, QT and QRT treatments result in increased exosome release, suggesting that free cholesterol efflux might occur through this pathway (Supplementary Figure 2). Interestingly, addition of free cholesterol decrease lysosome swelling (Figure 5C), rescued tumor cells viability (Figure 5D), and the LDLR overexpression was not observed (Figure 5E). LXR was not activated after CQ treatment (Figure 5E). Previous results showed cell death in Q, QR, QT and QRT treatment from ROS production (Figure 3B), while the additional free cholesterol significantly decreased ROS in in GBM8401 after Q, QR, QT, and QRT treatment (Figure 5F). Similar result was observed in U87MG (data not show). The results indicated that cell death of QRT treatment closely related to cholesterol depletion and ROS production. Cell membrane associated NADPH oxidase activation is suspected [34, 35].

### The triple combination treatment effectively treats GBM orthotropic xenografts

**Figure 6:**
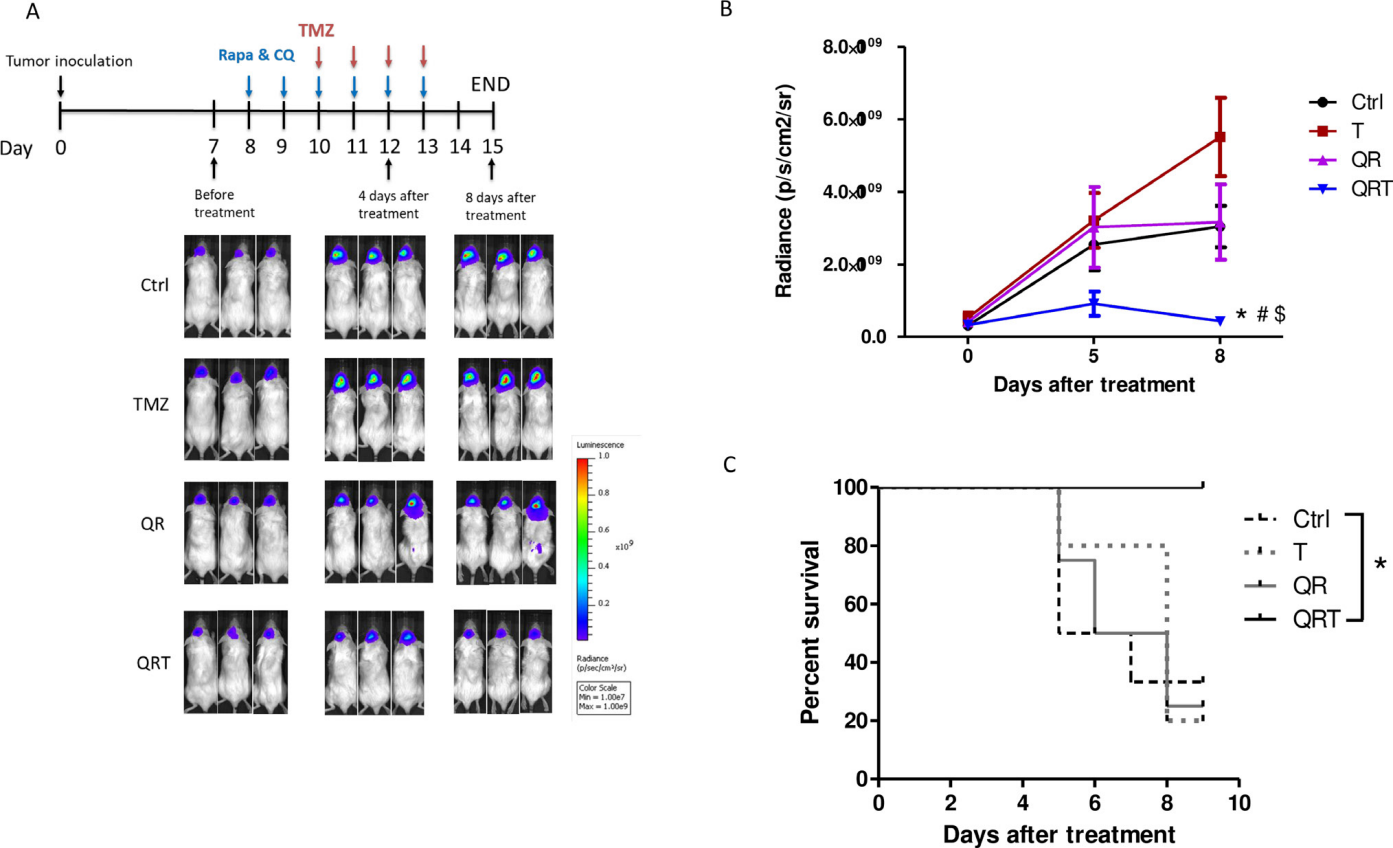
Decreased tumor growth with *in vivo* treatment of GBM xenograft model monitored by bioluminescence imaging (**A**) The treatment schedule of GBM xenograft. GBM8401-luc cells (2 × 10^5^) were injected stereotactically implanted into a single defined left hemisphere location (6 weeks old, *n* > 4) and the bioluminescence signal was monitored by the *in vivo* imaging system at the indicated days after inoculation. Seven days after tumor implanted received no treatment (control), treatment with TMZ (T, 50 mg/kg i.p.), CQ (50 mg/kg i.p.) + Rapa (5 mg/kg i.p.) (QR) and QRT. Tumor cells spread rapidly in the untreated control mice. When the intracranial brain tumors were treated with TMZ alone or QR, in both cases are similar patterns of untreated control. Tumor treatment by QRT significant slowed the growth of the tumors by day 4 after implantation. (**B**) Mice of QRT treatment significantly inhibit tumor growth compared with control, TMZ, and QR treatment. (**C**) QRT treatment was prolonged survival time. Statistical significance: Control *vs* QRT (^*^), T *vs* QRT (^#^), QR *vs* QRT (^$^).

To further evaluate the efficacy of QRT triple treatment, NOD/SCID mice bearing intracranial GBM8401 xenografts were treated. After 7 days implantation GBM8401, intra-peritoneal injections of TMZ alone, QR or QRT were performed from day 8 to day 15. Tumor progression was evaluated with IVIS imaging over time as Figure 6A mentioned. A representative photo image was presented in Figure 6A. Tumor cells rapidly proliferate in untreated controls. Inhibition of tumor cell growth is observed QRT treatment, and there was significantly decrease tumor growth in QRT triple treatment (Figure 6B). QRT treatment was prolonged survival time comparison with other treatments (Figure 6C). We sacrificed mice on day 15 based on IACUC recommendations, loss of 25–30% of initial body weight should be considered as death with euthanasia administered according to institutional guidelines.

## DISCUSSION

This study suggests that GBM is susceptible to the induction of cholesterol depletion related to the lysosome dysfunction. High cholesterol turnover is essential for the survival of GBM cells under stress. In comparison to mTOR inhibitor (Rapa) treatment alone, the combined Rapa and lysosome inhibitor (CQ) with or without TMZ therapy significantly increased apoptotic cell death through mitochondria damage before cathepsin released from lysosome in one type of GBM cells (GBM8401), while the addition of TMZ decidedly increased apoptotic cell death through cathepsin induced cell death in other type of cells (U87G). We observed that triplet combination (QRT) treatment is synergistic in all type of GBM cells through depleted membrane cholesterol content along with ROS formation and the cytotoxicity can be reversed by external added cholesterol or NAC antioxidant.

Malignant glioma has a high lipid anabolism phenotype, enabling rapid cell proliferation [36–38]. Lipids, such as phospholipids, fatty acids, cholesterol, triglycerides and cholesterol esters, and sphingolipids, are important components of cells including the caveolin membrane system [15]. Most brain cholesterol is synthesized *de novo* in normal astrocytes, because cholesterol in the bloodstream cannot be transported across the blood brain barrier. GBM cells rely on uptake of exogenous cholesterol from neighboring cells [8]. Increased cholesterol esterification and storage in LD, in addition to increased LDL uptake are beneficial for GBM [10]. Cellular cholesterol levels are controlled by biosynthesis, cellular LDL uptake through LDL receptors, phagocytosis and efflux from cells. Lipid breakdown leads to release of free fatty acids that sustain β-oxidation in the mitochondria. As shown in Figure 2A through 4C, we observed an autophagy activation as evident by suppression of p70S6K phosphorylation with increased ROS production and a decreased LD after Rapa treatment. CQ markedly increased LC3B-II conversion, p62 accumulation, lysosome clumping and LD accumulation, which indicated of lysosome blockage. Rapa treatment mimicked a state of nutritional starvation, the simultaneous combined autophagy inducer and inhibitor as QR treatment resulted in marked lysosomal swelling and LD accumulation. Because cells depended on LD fueled fatty acids for survival during nutrient deprivation [9]. Lippincott-Schwartz *et al.* showed that fatty acids are released from LDs by lipolysis and taken up by fused LDs with mitochondria to support oxidative respiration [39]. However, does fatty acid move from LD due to cytoplasmic lipase-mediated lipolysis instead of lipophagy, and how fatty acid trafficking after QR or QRT treatment causes mitochondria change are interesting issues for further study [39, 40].

In GBM cells, endolysosomes are required for digesting extracellular lipoproteins after endocytosis, and autophagy-mediated LD degradation also required lysosomes for lipid mobilization. The high dependence of GBM cells on lysosomes for lipid metabolism, coupled with their limited autophagy capacity makes GBM cells more vulnerable to CQ treatment-related cholesterol deficit. Maintaining cellular free cholesterol levels is essential for cell membrane maintenance, viable tumor cell morphology and lysosome function. CQ blocks cholesterol extraction from LDL contained in endosomes and from lipid droplets by inhibiting lysosomal function. CQ has long been known to increase rapid retrograde LDL exocytosis and delay the hydrolysis of cholesterol esters [39, 41]. Higher cholesterol efflux in GBM cells through increased exocytosis instead of ABCA1 transporter are evident from exosome release after QT treatment. Since *de novo* cholesterol synthesis is also depressed with addition of Rapa-mediated SREBP down-regulation, adequate cholesterol reserves are not available to tumor cells. Triple QRT treatment results in accumulation of cholesterol esters in lipid droplets and failure of cholesterol mobilization upon ester hydrolysis, which significantly reduces availability of free cholesterol in tumor cells. Cellular cholesterol content directly influences lysosomal stability, and the increased lysosomal membrane permeability results in cathepsin release and increases apoptosis. The production of ROS is closely associated with cholesterol levels. NADPH oxidase is one of the main ROS-generating enzymes present in lipid rafts and both cholesterol content [34]. As shown in Figure 5, cholesterol repletion abolished the ROS production and reversed the cytotoxicity from QRT treatment.

The Warburg effect of utilizing aerobic glycolysis as the primary supplier of ATP is found in GBM and many cancers. However, oxidative phosphorylation (OXPHOS) function is very important in GBM cells. A prior study showed that glioma stem cells are less glycolytic and use mitochondria for glucose oxidation *in vivo* [42–44]. Recent studies of orthotropic GBM xenografts demonstrated that GBM uses both glycolysis and mitochondrial oxidation for glucose catabolism. As shown in Figure 3D, the Rapa and CQ double combination has dual inhibitory action on OCR and ECAR, while the mitochondria function was most severely impaired. The decreased ATP supply further increases cellular dependence on autophagy functions. Simultaneous targeted disruption of multiple important cellular metabolism pathways may synergize to result in ‘synthetic lethality’.

A recent phase II GBM clinical trial failed to show benefit after addition of everolimus, a Rapa analogue, to the standard TMZ regimen [45]. Activation of autophagy alone seems to be insufficient to reverse GBM therapeutic resistance. Our proposed approach of adding the autophagy inhibitor CQ appears to be more promising. A small randomized trial demonstrated the survival benefit of adding CQ to a combination of radiotherapy and lomustine in newly diagnosed GBM patients [46]. Rapa and CQ has been reported to own the ability to penetrate blood-brain barrier [47, 48]. The present study demonstrates that cholesterol depletion may be a potential therapeutic mechanism of CQ that overcomes limitations in caspase-dependent apoptotic cell death. Therefore, this study suggests that triple therapy of Rapa, CQ and standard TMZ-RT treatment would be a promising therapeutic strategy to test in clinical trials for GBM patients.

## MATERIALS AND METHODS

### Cell culture

GBM8401 cells, kindly provided by Professor Hsin-Ell Wang (Department of Biomedical Imaging and Radiological Sciences, National Yang-Ming University, Taiwan). U87MG cells, M059K cells, and Hs683 cells were obtained from the American Type Culture Collection (ATCC, Manassas, VA). U87MG were cultured in MEM with 1 mM Na pyruvate, 10% FBS and antibiotics (100 U/ml penicillin and 100 μg/ml streptomycin). GBM8401 were cultured in RPMI with 10% FBS and antibiotics (100 U/ml penicillin and 100 μg/ml streptomycin). M059K were cultured in MEM with 10% FBS, 0.1 mM NEAA, 1 mM sodium pyruvate. Hs683 were cultured in DMEM/F12K with 10% FBS, 0.05 mM MEAA, 0.5 mM sodium pyruvate. Cells were grown in a 5% CO_2_ incubator at 37°C.

### Cell proliferation assay

Cell cultures was performed by seeding at a density of 1.0 × 10^5^ cells/well in 96-well round-bottom plates (Falcon, UK) containing 200 μL of medium. Various doses of chemotherapeutic drugs and CQ, in combination with Rapa (at different molar ratios) were added after tumor cells reached 1 × 10^6^ density. Treated tumor cells were maintained for two days at 37°C in humidified 5% CO_2_. The rate of cell proliferation was measured using an MTS assay (CellTiter 96 aqueous one-solution cell proliferation assay; Promega, WI, USA). 40 μL of CellTiter 96 aqueous one-solution were added to each well. After 4 h of incubation, the UV absorbance of the solution was measured at a wavelength of 490 nm. All MTS assays were performed in triplicate.

### Apoptosis assay

Apoptosis was assayed using an Annexin V Apoptosis Kit (BD Pharmingen, CA, USA) according to manufacturer instructions. Briefly, tumor cells were washed three times with PBS, then immediately analyzed for apoptosis using Annexin V/PI (propidium iodide) staining. Washed cells were supplemented with 1% BSA, then directly stained with 10 μL of PI and 2.5 μL Annexin V-FITC after adding 222.5 μL of binding buffer. Immediately following a 10 min incubation period in the dark on ice, the cells were analyzed by flow cytometry. The percentage of positive cells was determined by using a BD Accuri™ C6 and BD Accuri™ C6 software (Becton Dickinson, Mountain View, CA, USA).

### Western blot analysis

For protein analysis, cells were lysed for 5 min at room temperature in a buffer composed of 150 mM NaCl, 50 mM Tris (pH 8.0), 5 mM EDTA, 1% (v/v) Nonidet p-40, 1 mM phenylmethylsulfonyl fluoride, 20 μg/mL aprotinin, and 25 μg/mL leupeptin (Sigma). The total protein concentration of lysates was measured using the Bio-Rad protein assay (Bio-Rad, Hercules, CA, USA). Cell lysate (100 μg) was electrophoresed on a 12% polyacrylamide gel and the proteins were transferred to an Immobilon-P PVDF membrane (Millipore, Bedford, MA, USA), which was then blocked for 2 h at room temperature in PBS containing 0.05% Tween 20 and 10% nonfat milk. The membrane was then incubated with antibodies against β-actin (Sigma, 1:10000), GAPDH (Sigma, 1:10000), LC3 (Novus Biologicals Inc., Littleton, CO, 1:10000), SQSTM1/p62 (MBL international, 1:1000), PARP (Cell Signaling Technologies, 1:1000), phospho-mTOR, phospho-Akt (Ser 473) (Cell Signaling Technologies, 1:1000), phospho-p70S6K (Cell Signaling Technologies, 1:1000), ABCA1 (Abcam, 1:1000), LDLR (Abcam, 1:5000), SREBP1 (BD, 1:1000), SREBP2 (BD, 1:1000), CHOP (Cell Signaling Technologies, 1:1000) and GRP78 (Cell Signaling Technologies, 1:1000) overnight at 4°C in PBS containing 0.05% Tween 20 and 5% nonfat milk, followed by incubation for 1 h at room temperature with horseradish peroxidase-conjugated secondary antibodies (Jackson ImmunoResearch Laboratories, West Grove, PA, USA) in the same buffer. Blots were developed using a chemiluminescent detection system (ECL; GE Life Science, Buckinghamshire, UK).

### Determination of mitochondrial membrane potential (MMP)

MMP was measured using a BD MitoScreen kit (JC-1, BD Biosciences) according to the manufacturer's instructions. Briefly, 1 × 10^6^ cells were washed, resuspended in 500 μL of JC-1 working solution, then incubated at 37°C for 15 min. Following staining, cells were washed twice and resuspended in 500 μL of assay buffer. Cells were immediately analyzed using a BD FACSCalibur flow cytometer. Live cells were gated and analyzed.

### Measurement of ROS production

Intracellular ROS was detected by 2′,7′-dichlorofluorescein diacetate (DCFDA, Sigma). Cells were washed with PBS and incubated with DCFDA (0.25 μM) for 10 min at 37°C. The florescence intensity was detected using BD Accuri™ C6 and BD Accuri™ C6 software. Untreated cells were used for normalization.

### Evaluation of oxygen consumption and extracellular acidification rate of metabolic parameters

Metabolic responses of GBM8401 cells were evaluated with an Extracellular Flux Analyzer (XFe24; Seahorse Biosciences, North Billerica, MA) according to manufacturer instructions. The Extracellular Flux Analyzer measures oxygen consumption and extracellular acidification rates (ECAR) of a defined number of cells in a small culture media volume in real time, and monitors cellular responses to drug treatment. In brief, 5 × 10^4^ cells were seeded in XFe24-well plates and incubated overnight at 37°C in a 5% CO_2_ humidified atmosphere, followed by treatment with indicated drug concentrations for 2 hr. After 2 hr, mitochondrial respiration was measured by oxygen consumption rate (OCR), and glycolysis was evaluated by ECAR after injecting the following inhibitors of mitochondrial respiration: oligomycin (inhibitor of ATP synthase, 1 uM), FCCP (uncoupling factor), Antimycin A/Rotenone (inhibitor of mitochondrial complex I of the ETC), and 2-deoxyglucose (2-DG; inhibitor of hexokinase). Basal OCR and ECAR were measured, as well as the changes in oxygen consumption caused by adding the above metabolic inhibitors.

### Immunofluorescence

U87MG and GBM8401 cells were fixed with 4% paraformaldehyde for 15 min at room temperature and the samples were twice-washed with PBS. Samples were then incubated for 10 mins with PBS containing 0.25% Triton X 100 followed by PBS rinse three times for 5 mins. After incubating the cells with 1% BSA in PBST for 30 min, they were probed with a mixture of two primary antibodies, Cathepsin B (ab58802, Abcam, 1:500), and LAMP1 (#9091, cell signaling, 1:500) in 1% BSA in PBST for 1 hr at room temperature. Images were captured using a Zeiss LSM800 with AirScan laser confocal microscope.

### Lipid and cholesterol staining

U87MG and GBM8401 cells were fixed with ice-cold methanol for 10 min and washed thrice with PBS, followed by incubation with 1 ml of 1.5 mg glycine/ml PBS for 10 min at room temperature. Free cholesterol in cells was stained with 1 ml of Fillipin (Sigma-Aldrich, St. Louis, MO, USA) working solution (0.05 mg/ml in PBS/10% FBS) for 2 h at room temperature. For LD staining, fixed monolayers with 4% paraformaldehyde were incubated with 10 μg/ml BODIPY 493/503 (Molecular Probes, Eugene, OR, USA) for 30 min at room temperature. Slides were washed, stained with Hoechest (Cell signaling, Technology, Beverly, MA, USA) and visualized using fluorescent microscopy (Zeiss LSM800 with AirScan laser confocal microscope).

### Intracranial glioma orthotropic xenograft model

Human brain malignant glioma cells from the GBM8401 cell line were transformed with the luciferase gene (GBM8401-luc). All procedures were performed according to the guidelines approved by the Animal Care and Use Committee of the National Yang-Ming University. Male 6- to 8-week-old NOD/SCID mice were anesthetized via intraperitoneal administration of pentobarbital at 40 mg/kg body weight. Their heads were shaved above the nape of the neck, scrubbed with Betadine/alcohol, and immobilized in a Cunningham Mouse/Neonatal Rat Adaptor stereotactic apparatus (Stoelting, Wood Dale, IL, USA). A 5-mm skin incision was made at the sagittal suture, then a burr hole was created, and 2 × 10^5^ GBM8401-lu cells in 2 μl of culture medium were injected stereotactically implanted into a single defined left hemisphere location (0.14 mm anterior and 2.0 mm lateral to the bregma) of each mouse brain at a 3.5 mm depth. The skull burr hole was then sealed with bone wax and the wound was flushed with iodinated alcohol. Biophotonic imaging was used to confirm tumor engraftment.

### *In vivo* drug treatment

Tumor size was quantified by analyzing biophotonic images obtained 7 days after tumor cell implantation. Cohorts of 5 mice per experiment with approximately equivalent tumor bioluminescence were divided into four groups: 1) control (saline,i.p.), 2) TMZ alone (50 mg/kg, i.p.), 3) Chloroquine (50 mg/kg, i.p.) + Rapamycin (5 mg/kg, i.p.), 4) TMZ + Chloroquine + Rapamycin (three-drug, i.p.). Each group was treated for six days, and TMZ was administrated from day 3 to 6.

### *In vivo* imaging

After anesthesia with isoflurane (1.5 l/min oxygen in 4% isoflurane), each mouse was injected intraperitoneally with 4.29 mg of freshly prepared luciferin substrate suspended in phosphate-buffered saline (PBS) and imaged 10 min later using the Xenogen IVIS imaging system (Xenogen, Palo Alto, CA, USA), with a 1-min acquisition time in small-bin mode. Luciferase activity was quantified within a region of interest that encompassed the head of the mouse using Living Image Software (Xenogen, Palo Alto, CA, USA).

### Statistical analysis

All statistical analyses were performed using Prism 4 (GraphPad Software, Inc., La Jolla California USA, www.graphpad.com). The experimental and control groups were compared using an unpaired two-tailed Student's *t* test. Statistical analysis was performed at the *P* < 0.05 (denoted as ^*^).

## SUPPLEMENTARY MATERIALS FIGURES AND TABLES


