# Supplementary material for: Temozolomide, sirolimus and chloroquine is a new therapeutic combination that synergizes to disrupt lysosomal function and cholesterol homeostasis in GBM cells
Source: Oncotarget. 2018 Jan 3;9(6):6883–96. doi: 10.18632/oncotarget.23855 (PMC5805523; doi:10.18632/oncotarget.23855)
Supplement: Supplementary file 1 [file oncotarget-09-6883-s001.pdf]

Temozolomide, sirolimus and chloroquine is a new therapeutic combination that synergizes to disrupt lysosomal function and cholesterol homeostasis in GBM cells

SUPPLEMENTARY MATERIALS

MGMT

DNA Extraction/Bisulfite: Genomic DNA extraction for all bisulfite conversion was done using the Favor- Prep™ Tissue Genomic DNA Extraction Mini-Kit (Favorgen) as recommended by the manufacturer. For validation of shore regions, 1 ug of genomic DNA from each sample was bisulfite-treated using EZ DNA Methylation-Gold Kit (Zymo Research) according to the manufacturer's suggestion. Up to 2000 ng (at least 1 µg) of DNA per sample are bisulfite treated to obtain adequate converted DNA for quantitative real-time polymerase chain reaction (Q-PCR). Methylation-specific PCR: MSP analysis was performed with the following specific primers designed to distinguish methylated (MetMGMT) from unmethylated DNA (UnmetMGMT) (5′–3′):
UnmetMGMT F: TGTGTTTTGGATATGTTGGGATAGT,
UnmetMGMT R: AACTCCACACTCTTCCAAAAACAA,
MetMGMT F: GCGTTTCGACGTTCGTAGGT-3′ and
MetMGMT R: CACTCTTCCGAAAACGAAACG. COL2A1 gene was used as internal control (5′–3′); F: TCT AACAATTATAAACTCCAACCACCAA, R: GGGAAG ATGGGATAGAAGGGAATAT-MSP using SYBR Green Master mix was performed using PCR Core Reagents (Applied Biosystems) with 1 µl of bisulfite-treated DNA. Final concentrations in a volume 20 µl were 10 µl Master mix, 0.6 µM of primers. PCR is carried out on an ABI Prism StepOne Real-time PCR system (Applied Biosystems) with the following amplification program: 10 min at 95°C followed by 40 cycles of 95°C for 15 s and 60°C for 1 min.

Exosome measurement

Exosome immuno-adsorption and detection was performed with FACS. Cell culture supernatants were pre-cleared by different centrifugations at 350 rpm for 10 min, 2,000 rpm for 15 min and filtered with .22 µm filter. 10 ml of pre-cleared supernatants were incubated with anti-CD63 magnetic beads (from Biovesicle Inc.) overnight at 4°C. Extracellular vesicles (EVs)-beads complex was washed twice in buffer (Biovesicle Inc.) at room temperature. EVs-beads complex was incubated with PE-conjugated anti-CD81 antibody for 30 min at 4°C. Antibody-stained EVs-bead complexes were acquired on BD™ LSR II (BD Biosciences) and data analyzed with FlowJo software (Tree Star, Ashland, OR).

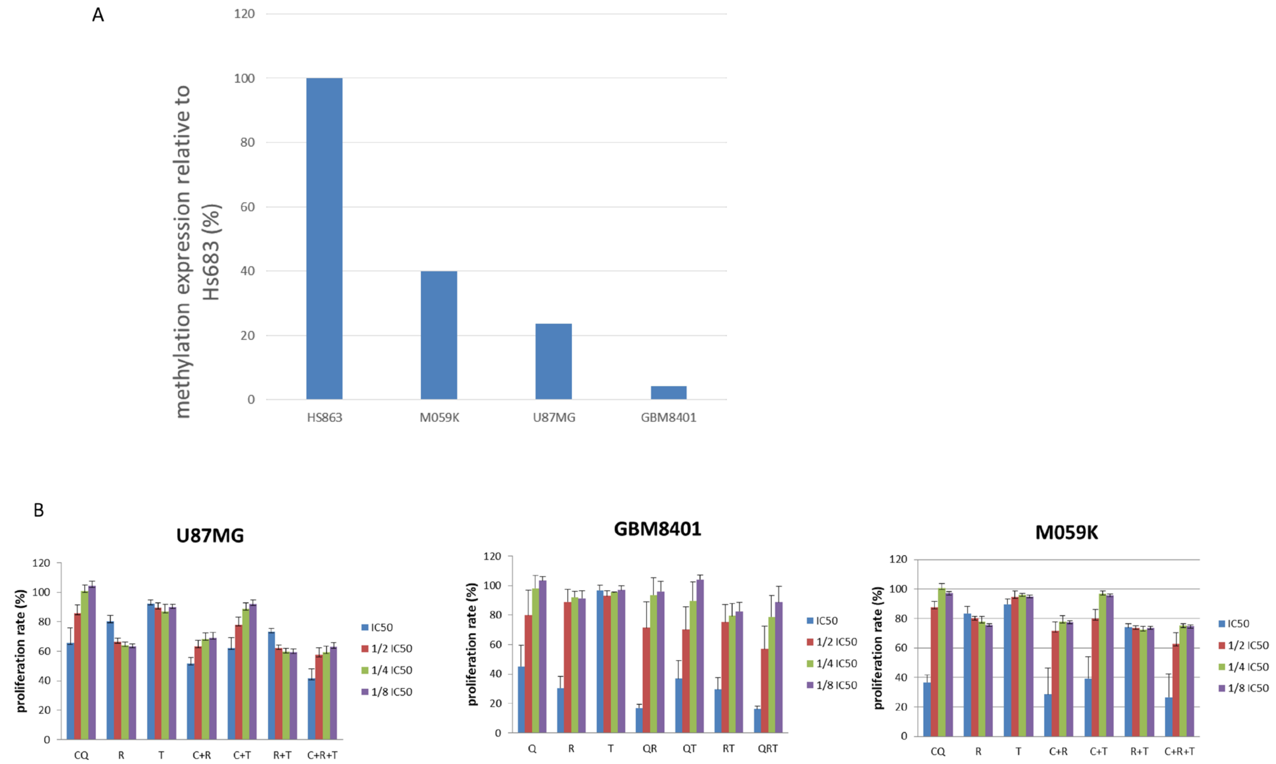

Supplementary Figure 1: (**A**) The expression of MGMT methylation in four cell lines. (**B**) All GBM cell lines were cultured as following treatment to assay dose-response analysis (IC50: GBM8401 Q 40/R 12.5/T 400 µM 72 h, M059K Q90/R 20/T 400 µM 72 h, U87MG Q 40/R 10/T 400 µM 72 h).

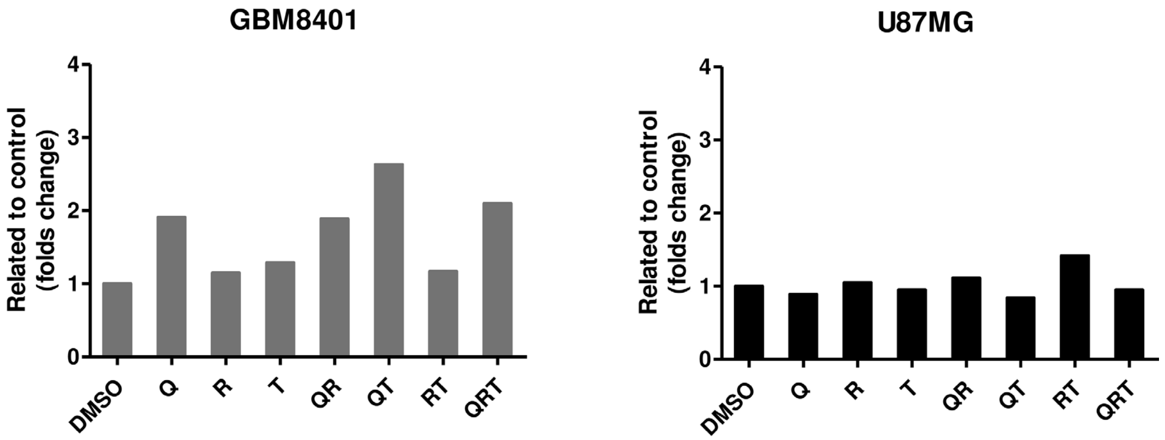

Supplementary Figure 2: CQ and TMZ or Rapa and TMZ synergistic increase exosome release. After GBM8401 and U87MG culture with CQ, Rapa and TMZ combination for 24 hr, supernatant was collected for exosome detection.
